# Progress of Epidemics in Twenty-Seven Towns and Districts

**Published:** 1856-07

**Authors:** 


### PROGRESS OF EPIDEMICS.

### LOCAL REPORTS OF EPIDEMIC AND ENDEMIC DISEASES

*During the Months of March, April, and May,* 1856.

| Place. | County. | Lat. | Long. | Observer. |
|---|---|---|---|---|
| Teignmouth - | Devonshire - | 50.32 N. | 3.29 W. | W. C. Lake, Esq. |
| Odiham - - | Hampshire - | 51. 8 N. | 1. 3 W. | J. M'Intyre, M.D. |
| Canterbury - | Kent - - | 51.17 N. | 1. 4 E. | G. Rigden, Esq.<br>James Reid, Esq. |
| Chatham - | Kent - - | 51.24 N. | 0.14 E. | F. J. Brown, M.D. |
| Putney - - | Surrey - - | 51.28 N. | 0. 3 W. | R. H. Whiteman, Esq. |
| Up. Holloway | Middlesex - | 51.32 N. | 0.03 E. | W. B. Kesteven, Esq. |
| Wanstead - | Essex - - | 51.32 N. | 0. 2 W. | F. Collins, M.D. |
| Saffron Walden | Essex - - | 52. 3 N. | 0.12 E. | T. Spurgin, Esq.<br>H. Stear, Esq. |
| Bedford - - | Bedfordshire - | 52. 8 N. | 1.51 W. | T. H. Barker, M.D. |
| Sharnbrook - | Bedfordshire - | 52.12 N. | 0.40 W. | R. S. Stedman, Esq. |
| Newpt.Pagnell | Buckinghams. | 52.10 N. | 0.41 W. | G. O. Rogers, Esq. |
| Wellingbro' - | Northamptons. | 52.20 N. | 0.40 W. | B. Dulley, Esq. |
| Thetford - | Norfolk - - | 52.26 N. | 0.45 E. | H. W. Bailey, Esq. |
| Wisbeach - | Cambridgesh. | 52.39 N. | 0. 5 E. | W. H. Hole, Esq. |
| Pontesbury - | Shropshire - | 52.43 N. | 2.50 W. | Wm. Eddowes, Esq. |
| Nottingham - | Nottinghamsh. | 52.50 N. | 1.10 W. | T. Robertson, M.D. |
| Burton-on-Trt. | Staffordshire - | 52.53 N. | 1.53 W. | S. Thomson, M.D. |
| Wrexham - | Denbighshire - | 53. 2 N. | 3. 1 W. | E. Williams, M.D. |
| Hawarden - | Flintshire - | 53.11 N. | 3. 2 W. | T. Moffat, M.D. |
| Lincoln - | Lincolnshire - | 53.12 N. | 0. 5 W. | S. Lowe, Esq. |
| Alford - - | Lincolnshire - | 53.15 N. | 0. 6 E. | R. U. West, M.D. |
| Gainsborough | Lincolnshire - | 53.23 N. | 0.47 W. | D. Mackinder, M.D. |
| Liverpool - | Lancashire - | 53.24 N. | 2.59 W. | Thos. Bickerton, Esq. |
| Bolton - - | Lancashire - | 53.35 N. | 2.19 W. | W.H. Pendlebury, Esq. |
| York - - | Yorkshire - | 53.58 N. | 1. 3 W. | W. Procter, Esq. |
| Wst. Auckland | Durham - | 54.45 N. | 1.40 W. | G. Todd, Esq. |
| Rothbury - | Northumberld. | 55.25 N. | 1.50 W. | E. C. Summers, Esq. |

### QUARTERLY STATEMENT—No. VI.

[*The dates denote that the disease appeared in the weeks then ending.*]

SCARLET FEVER.

Teignmouth..May 2 [May 9, 30
Odiham..All March, April 4, 18, 25,
Canterbury..All March, April 18, 25, May 9, 30
Chatham..Every week except Mar. 7
Up. Holloway..Mar. 28, April 4, 18
Wanstead..May 30
Saffron Walden..Mar. 28, May 16
Sharnbrook..Mar. 7-21, April 11-25,
Newport Pagnell..May 30 [May 16-30
Wellingborough..All Mar., April 4-11
Thetford..March 7
Wisbeach..All March, April 4
Pontesbury..All April, May 2-23
Nottingham..March 7, 28, April 4-11, May 30
Burton-on-Trent..March 7, April 11
Wrexham..Every week
Lincoln..April 18, 25, May 2
Gainsborough..April 18, 25, May 2
Liverpool..Every week except April 18
Bolton..All March, April 25, May 9, 30

MEASLES.

Odiham..Every week
Canterbury..Every week [9-23
Chatham..Mar. 7-21, April 4, 25, May
Up. Holloway..All April
Sharnbrook..Every week except Mar. 7 and May 16

Newport Pagnell..April 11-18, May
Wellingborough..April 18-25 [23-30
Nottingham..Every week
Wrexham..Every week
Alford..May 9
Liverpool..Every week except Ap. 18
Bolton..Mar. 28, April 11, all May
York..March 28, April 18

SMALL POX.

Canterbury..Mar. 7-21, Ap. 4-11, May 9
Chatham..Every week except Mar. 7
Putney..May 2 [and May 23
Bedford..March 7
Newport Pagnell..May 9
Wellingborough..Every week
Burton-on-Trent..May 23
York..Mar. 21, April 4-11, May 2, 16

HOOPING COUGH.

Teignmouth..Mar. 14, 28, all April,
Odiham..Mar. 21, Ap. 11 [May 2, 16
Canterbury..Every week [2-30
Chatham..All Mar., April 11-18, May
Putney..All March, April 4-18 [May
Up. Holloway..Mar. 7-14, Ap. 18, all
Wanstead..Mar. 14, April 11, May 16
Saffron Walden..Every week
Thetford..April 11-25, May 2
Wisbeach..Every week
Nottingham..Every week
Lincoln..March 7-21
Alford..March 7-14, May 9, 23-30
Gainsborough..All March, April 4-18
Liverpool..Every week
Bolton..Mar. 21-28, April 4, May 9-30
York..Mar. 7, 28, Apr. 11-25, May 2, 23
Rothbury..Mar. 28, Apr. 4-11, May 30

CROUP.

Canterbury..March 14-21, April 11,
Chatham..Mar. 7, April 18 [May 9
Putney..May 2
Upper Holloway..March 21
Bedford..April 11
Sharnbrook..March 28, April 4
Wisbeach..March 21, 28 [May 9-23
Nottingham..Mar. 14-28, April 4-18,
Wrexham..May 2, 9
Gainsborough..April 25 [May 2, 9
Liverpool..March 28, April 4, 11, 25,
Bolton..March 7, 21

CATARRH.

Teignmouth..Every week except May
Odiham..Every week [23
Canterbury..All March, May 2
Chatham..Every week
Putney..All Mar., May 16 [May 2-16
Up. Holloway..March 28, April 4-11,
Wanstead..March 7
Saffron Walden..Every week
Bedford..April 11
Newport Pagnell..All April, May 2-9
Wellingborough..All March and Apr.
Thetford..March 7, 14, 28, April 4, 11, 25, May 2-16
Wisbeach..All Mar., Apr. 4, May 23-30
Pontesbury..All March [May 2, 16-30
Nottingham..All March, April 11-25,
Burton-on-Trent..All Mar. and April
Wrexham..April 11-25, May 2-9
Alford..March 21
Gainsborough..Every week
Liverpool..Every week
Bolton..All Mar., April 25, May 2-9
York..Mar. 14, 28, April 25, May 2
Rothbury..May 9-30

INFLUENZA.

Teignmouth..Every week except Apr.
Odiham..Mar. 7, 21, Apr. 11-18 [4 & 25
Chatham..All March, April 4, 11, 25, May 16-23
Putney..All Mar., Apr. 4-18, May 2
Wanstead..Mar. 7-14, Apr. 4-11, 25, May 9-16
Saffron Walden..Every week
Bedford..April 11-25, May 9-23
Sharnbrook..All March and April
Newport Pagnell..All April and May
Wellingborough..Every week
Thetford..March 7-14, 28, April 4-18, May 2-16
Wisbeach..May 2-16 [May 2-16
Pontesbury..March 21-28, all April,
Nottingham..Mar. 7, Apr. 4, May 9, 30
Wrexham..Mar. 21-28, all April, May
Hawarden..April 4 [2-9
Lincoln..March 7-14 [and 23
Liverpool..Every week except May 16
York..Mar. 14, Apr. 11-18, May 2-23
West Auckland..Every week

CHOLERA.

Liverpool..May 30

AGUE.

Canterbury..Mar. 7-14, all April, May
Chatham..Every week [9, 23
Bedford..April 18, May 2, 16, 30
Sharnbrook..May 2-16, 30
Newport Pagnell..May 2
Wellingborough..April 25, all May
Thetford..Mar. 21-28, Apr. 25, May 23
Wisbeach..Every week
Alford..May 30

ERYSIPELAS.

Teignmouth..March 21-28, April 18
Odiham..Mar. 14, April 4 [May 2
Canterbury..Mar. 7, 21-28, Apr. 11-18,
Chatham..Mar. 14-28, Apr. 4, 25, May
Putney..April 25 [2, 9
Saffron Walden..April 18
Bedford..May 9
Sharnbrook..May 30
Newport Pagnell..April 11, May 2

Thetford..Mar.14-28, Apr.11, 25, May
Wisbeach..Mar. 21-28, Apr. 4 [16
Pontesbury..Mar.7-21, Apr.18, May 23
Nottingham..April 18, May 9-23
Burton-on-Trent..Mar. 14, 28, Apr. 11
Hawarden..Mar. 21, April 4
Lincoln..May 23, 30
Alford..May 30
Gainsborough..April 11-25
Liverpool..April 11, May 9, 30
Bolton..March 14, 28, all April
West Auckland..May 16-30
Rothbury..May 2

REMITTENT FEVER.

Teignmouth..All April, May 23-30
Odiham..Mar. 7, Apr. 4-11, May 2, 30
Canterbury..March 14-21
Putney..All March, May 9, 23-30
Upper Holloway..April 18-25
Saffron Walden..April 4-11, May 9-30
Sharnbrook..Mar. 14-28, April 4, 18, May 9
Newport Pagnell..All April, May 2
Thetford..Mar. 14, May 2, 23 [16-30
Wisbeach..Mar. 28, all April, May
Nottingham..May 23
Burton-on-Trent..April 18
Alford..May 16-23 [May 9, 30
Liverpool..All Mar., Apr. 4, 11, 25,
Bolton..April 18-25, May 2
York..Mar. 28, Apr. 4-11, May 2-16

DIARRHŒA.

Teignmouth..Mar. 21-28, Apr. 4, 11,
Odiham..May 9, 30 [25, May 2, 9, 30
Canterbury..Mar.7-21, all April & May
Chatham..Every week except May 9 and 23
Putney..All March, April 4, 18
Upper Holloway..Mar. 28, April 4
Wanstead..Mar. 7, 21, April 4
Saffron Walden..Apr. 25, May 9-30
Bedford..March 7, 21, April 4, 18, May 9-30 [May 21, 30
Sharnbrook..All Mar., Apr. 4, 18, May
Newport Pagnell..All April, May 2, 9, 23, 30
Wellingborough..All March and Apr.
Thetford..All March, Apr. 11, 25, all May [2-9
Wisbeach..Mar. 14-28, Apr. 4-11, May
Pontesbury..March 7-14, April 25, May 16
Nottingham..Apr. 18-25, May 9-30
Burton-on-Trent..April 25, May 9
Hawarden..All Mar. and Apr., May 16
Alford..Mar. 21, May 16-30
Gainsborough..All March and April, May 2, 9 [May
Liverpool..Mar. 14-28, April 4, 25, all
Bolton..Apr. 4-11, 25, May 2-9, 23
York..March 21, April 4
Rothbury..April 11, May 2-16

DYSENTERY.

Odiham..Mar. 14-21, May 9-16
Chatham..May 9
Saffron Walden..April 25
Bedford..May 16, 23
Newport Pagnell..Apr. 11-25, May 2-9
Thetford..May 23
Nottingham..March 14, May 2, 23
Liverpool..May 23, 30
Bolton..April 25, May 2, 9

TYPHUS.

Teignmouth..March 21, April 11-25,
Canterbury..Every week [May 2
Chatham..Mar. 14-28, Apr. 4, 25, May
Putney..Apr. 18, May 9 [2, 9, 23
Upper Holloway..March 28
Wanstead..All March
Saffron Walden..Mar. 14-28, Apr. 18, 25, all May
Bedford..Mar. 7, 21, Apr. 4, 18, 25,
Sharnbrook..April 4 [May 2, 16
Thetford..Mar. 7, 25, April 4, 11, 25,
Pontesbury..Mar. 7, 14 [May 2, 30
Nottingham..March 14, May 2, 16
Gainsborough..Every week
Liverpool..Mar. 21-28, Apr. 4, 11, 25, all May
West Auckland..All March and Apr.,
Rothbury..Mar. 7-14 [May 2

PUERPERAL FEVER.

Odiham..May 9
Chatham..April 11, May 2, 9
Bedford..April 25
Thetford..March 7, 28
West Auckland..Mar. 7-21, May 2-23

CARBUNCLE.

Odiham..May 30
Canterbury..Every week
Chatham..April 25
Putney..May 9
Saffron Walden..May 2, 16
Sharnbrook..April 4, 18
Newport Pagnell..May 23, 30
Thetford..Mar. 14, April 11, May 23
Wisbeach..March 7, 14
Burton-on-Trent..Mar. 14
Liverpool..Mar. 7-14, 28, April 18-25, May 2, 9, 23, 30
Bolton..March 28, April 11
York..May 2
Rothbury..April 18

NEURALGIA.

Wrexham..Every week

RHEUMATIC FEVER.

Wrexham..March 28, April 4, 11, 25, all May

### ADDITIONAL OBSERVATIONS.

*Teignmouth.*—Mr. Lake writes: "The easterly winds, commencing on Feb. 28, blew, with the exception of March 5 and 19 to 21, till April 2, and principally from the N.E.; from this date till the 13th the wind was westerly, chiefly S.W.; from April 13 to May 11, with the exception of two or three days, it was again from the east, variably N.E. and S.E.; from May 11 to 28 westerly winds prevailed. No rain fell from Feb. 20 till March 16, a period of twenty-five days; from 16 to 18, 2·071 inches fell, but only 0·022 from that date till April 1. The mean barometric pressure for March was 30·075; mean maximum temperature, 47·2; mean minimum temperature, 36·9; mean humidity, 78; mean ozone, 3·1. The first half of April was showery, but not warm; the latter half coming in with very strong easterly winds, was at first bleak, then fine and bright, the sun being hot and the air cold. Mean barometric pressure for April was 29·686; mean maximum temperature, 53·9; mean minimum temperature, 40·9; mean humidity, 79; mean ozone, 5·2; total rain fell, 3·483 inches. The latter part of April and the month of May presented much variation of temperature; the first days of May were very cold; for a few days in the middle of the month the temperature was almost that of summer; it again declined considerably in a few days time, and the latter part of the month was showery and close, but not pleasantly warm.

"The most prevalent complaints were catarrh, also pleurisy and hooping-cough; one case of the last-named disorder was followed by extensive and fatal gangrene of the right cheek, involving its whole substance. Cases of chicken-pox occurred at intervals through the quarter. The cases of fever, both in children and adults, partook of the bilious type, in some with a tendency to engorgement of the head. Conjunctivitis was of somewhat common occurrence in the middle weeks of April, and tonsillitis in the latter half of April and first three weeks of May. Several cases of glandular inflammation and abscess occurred in children, principally during the month of May."

*Odiham.*—Dr. M'Intyre says: "Disease has considerably increased during the last quarter. The cases of scarlet fever were acute. Several of them were followed by anasarca of a severe character, and in two of them death occurred. The case of puerperal fever terminated fatally. The labour was in every way natural; the patient (whose first labour this was) was a school-mistress, aged twenty-seven, with exhausted nervous energy from over-taxed brain. Bronchitis and pneumonia have been prevalent."

*Canterbury.*—Mr. Rigden writes: "During the past quarter, the prevailing epidemics have been measles, hooping-cough, and catarrh. Cases of typhus have been under treatment during each week; a few cases of scarlet fever continue to shew themselves occasionally; and there have been also some cases of small-pox, but no case of this disease has come under my observation during this period. The bills of mortality for Canterbury show deaths from measles, hooping-cough, erysipelas, fever, scarlet fever, and dysentery in March; small-pox, measles, hooping-cough, fever, erysipelas, and diarrhœa in April; measles, scarlet fever, hooping-cough, catarrh, and fever in May."

Mr. Reid also has forwarded us the following valuable returns, which have been drawn up by the Epidemiological Committee of the East Kent and Canterbury Medical Society.

"*Abstract of Meteorological Observations for the Spring Quarter*, 1856.

| | *March.* Deg. | *April.* Deg. | *May.* Deg. |
|---|---|---|---|
| Lowest temperature in the day time .... | 32 | 37 | 42 |
| Highest .......................... | 47 | 53 | 60 |
| Mean .......................... | 38·86 | 50·58 | 50·70 |
| Lowest temperature in the night ...... | 24 | 29 | 34 |
| Highest .......................... | 44 | 47 | 55 |
| Mean .......................... | 35·23 | 40·33 | 44·54 |
| Lowest reading of the barometer ...... | 29·68 | 29·07 | 29·23 |
| Highest .......................... | 30·43 | 30·12 | 30·00 |
| Mean .......................... | 29·98 | 29·598 | 29·649 |
| Amount of rain in inches.............. | 0·37 | 3·27 | 4·19 |

Direction of wind (the number indicates the number of days certain winds prevailed)..*March*—N. 5, N.E. 9, E. 9, S.E. 1, N.W. 2, W. 4, S.W. 1; *April*—N.E. and N. 15, S.E. 2, S. 5, S.W. and W. 8; *May*—E. 8, N.E. 6, N. 4, N.W. 4, S., W., and S.W. 15.

"*March.* The comparison of this month with the months of the winter-quarter, shows that it was colder than either February or January, and that it ranged next to December in temperature. Vegetation, which began to push forwards in the latter part of February, was checked, and the few leaves that had appeared became brown and shrivelled at the edges. The only considerable fall of rain was on the 18th.

"*April.* On the 15th, 16th, and 17th, the valley of the Stour, and a portion of the flat part of the town, were flooded. This generally occurs when a N.E. wind blows strongly during, or immediately after, any considerable fall of rain. The inclination of the valley and the stream of water of the river is such, that it is probable that if all mill-dams and obstructions were removed, or even if the system of drainage were more complete, the city might not be liable to these injurious inundations. Rain fell during thirteen days this month, and on seven consecutive days; a severe thunder storm on the 30th.

"*May.* On the 8th, 9th, and 10th, the N.E. wind prevailing after a considerable fall of rain, the valley of the river was again flooded. Rain fell on twenty days during the month, at more frequent intervals than last month, the whole amount being greater by ·92 of an inch.

"The alternate impulse and check which has marked the progress of vegetation during the quarter has tended to produce a more equal degree of development; thus, the lime, beech, and elm trees, have pushed forth their buds and leaves each in its natural advance to a certain extent, but all more nearly together.

"*Abstract of the Returns of Zymotic Diseases during the Spring Quarter.*

"The number of cases of zymotic disease reported during the spring quarter has been 166. The greatest number of these were in the S.W., or upper part of the valley, district of the city; the next highest number in the N.W., or lower part of the valley, district; and in the third district, the E. or higher part, the smallest number. The mortality in the whole number was at the rate of 6·62 per cent., and was caused by measles, hooping-cough, scarlet fever, and small-pox.

"*Small-pox,* which appeared to have nearly worn out in the last month of the previous quarter, has furnished thirteen cases during the present quarter. Only three of the persons attacked were vaccinated, and these had it in a mild and modified form. Some of the remainder had the disease in a severe confluent form, and in one instance, a child three months of age, the disease was fatal.

"*Measles* have continued to prevail, but the monthly returns show that the number attended has gradually declined each month. The mortality has been at the rate of 4·38 per cent., chiefly from the complications of diseased lungs. One child, an emaciated subject, who had suffered from diarrhœa for eleven weeks previously, succumbed directly to the disease; another appeared to have died from phthisis quickly following the pneumonia from measles. Some instances of inflammation of the trachea, persisting for three or four days, were noticed; and several instances of irritation of the mucous membrane of the stomach and bowels were observed.

It is also mentioned, that in several infants and children under two years of age the ordinary coryzal symptoms were either absent or very slight, though the eruption was well defined.

"*Hooping-cough*, next to measles, has been most observed. Generally, only the worst cases of this disease come under treatment, so that it is difficult to attain a proper criterion of its prevalence, and the mortality is estimated at too high a proportion: thus in the cases observed, it has been at the rate of 18·18 per cent. The disease has been most frequently associated with, or consequent upon, measles. In one instance, death resulted from complication of tubercular disease of the mesenteric glands; another case, complicated with mesenteric disease, recovered. Two cases that recovered were complicated with diarrhœa.

"*Scarlet Fever* still occasionally appears; six cases are reported during the quarter, two of which were fatal, one from convulsions.

"*Fever*. No fatal case of this disease has been reported. Thirteen cases have been returned, two of which were complicated with severe head symptoms, and two with vomiting and bilious symptoms.

"*Ague* is generally more observed during the spring at Canterbury, though it is seldom absent altogether at other periods of the year. The type of the cases reported has been chiefly tertian, no case seems to have been protracted, though one is observed to have a tendency to relapse.

"*Diarrhœa* has been noticed by one or two observers as existing to a greater degree than usual at this period of the year. Disorders termed bilious seemed to be more prevalent than usual at the beginning of the spring. Mr. Rigden, the surgeon of the dispensary, observes, that during May several instances of furunculoid disease had presented themselves, and that there appears to be a tendency to diarrhœa amongst the population of Canterbury."

*Chatham.*—Dr. F. J. Brown writes: "During the last sanitary quarter (March, April, May) the wind was easterly on fifty-four days. The spring is backward, but vegetation has been making rapid advance for several weeks past. Scarlatina, measles, variola, and hooping-cough, have been epidemic; so also has been influenza. Erysipelas, ague, and diarrhœa, have prevailed. I know of three cases of metria that occurred in April and May; also of one case in a cow, for the week ending 30th May. Inflammatory affections of the mammæ have been numerous. Hæmorrhage in hooping-cough I have observed."

*Putney.*—Mr. Whiteman says: "Hooping-cough was the most prevalent disease of the zymotic class during the earlier portion of the quarter. The mortality, however, was not great. Many cases of remittent fever were treated during the month of March, especially in the undrained districts; and two cases of typhoid fever of a severe character occurred in April and May in the same localities. Diarrhœa was unusually prevalent in March, but the cases were none of them very severe or protracted, for the most part readily yielding to one or two doses of the nitrous acid mixture, employed by me so extensively and with such satisfactory results during the epidemics of 1849 and 1854. This town has been unusually free from severe and fatal disease since the construction of the main sewer in the High-street. It is the undrained localities that have yielded almost all the fatal cases for some time past. The sanitary measures that have been adopted under the Metropolis Local Management Act are beginning to manifest their good effects, particularly on the rate of mortality. When the sexton and parish clerk complain of a decrease in the amount of their fees, we may be pretty sure that some good influences are at work in improving the public health."

*Upper Holloway.*—Mr. Kesteven says: "The case of typhus is the first I have met with in Upper Holloway within a period of fifteen years. I have met with several unusually severe cases of varicella in May."

*Wanstead.*—Dr. Collins says: "The typhoid fever continued up to the last week in March, the total number of cases attended during January, February, and March, being seventy; the mortality, three. Since March the neighbourhood has been very healthy, excepting cases of influenza and hooping-cough of a mild kind, and one case of scarlatina."

*Saffron Walden.*—Mr. Spurgin says: "From the dry state of the atmosphere, notwithstanding the prevalence of north-easterly winds, the forepart of the quarter was healthy, but since that time indisposition has increased from the absence of solar heat, and augmented humidity of the air. Vegetation has been retarded from the same causes, but is now looking healthy and luxuriant."

Mr. Stear says: "Hooping-cough has been very prevalent; many adults have been suffering from it. I have attended several cases of infantile remittent fever lately."

*Bedford.*—Dr. Barker writes: "During the quarter ending May 30th, a few isolated cases of small-pox, croup, catarrh, and erysipelas, have occurred. We have had cases of ague, influenza, diarrhœa, dysentery, fever, and neuralgia, more particularly in the latter half of the quarter, but they have not been very numerous."

*Newport Pagnell.*—Mr. Rogers says: "The only case of small-pox that I am aware of was that of a young girl who *had* been vaccinated. It was severe; and the patient is marked. There is no idea as to the cause of infection. Some cases of measles have been very severe; but I have had no fatal case. I think the most prevalent diseases have been varieties of neuralgia. The wind has been principally N. and N.E. Latterly there has been a great deal of rain. Vegetation is luxuriant."

*Wellingborough.*—Mr. Dulley writes: "Of scarlet fever I have had but few fatal cases; in one, the efflorescence was very slight and transient, reappearing at intervals with threatening peritonitis; inflammation rapidly spread to the throat; sloughing of the fauces and tonsils occurred, and was followed by death on the fifth day. In the same neighbourhood there was another case with great pericardial oppression, much relieved by the full development of the rash; the recovery was slow. The cases of ague were confined to railway labourers."

*Thetford.* Mr. Bailey makes the following report. "The continuation of the mild weather, the undeviating state of the atmospheric pressure, together with the comparatively high temperature, and the little amount of rain and wind, during the commencement of this quarter, have occasioned less disease than usually occurs at this period. The specific epidemic diseases, which have so long visited us, have entirely left the town. Only one case of scarlatina has occurred, which was accidentally brought here by a poor family travelling the country, and which terminated fatally. The few cases of hooping-cough occurring in April seemed only the recurrence, from the cold weather, of former attacks, and which speedily gave way to medical treatment. Catarrhal affections have been very general, arising from the vicissitudes of temperature, and in some instances requiring assistance. Influenza has frequently occurred during the quarter, in old persons it has been fatal; and bronchitic affections with children have been extremely prevalent, requiring most active treatment, and fatal in three cases. Pleurodynia, in the labouring classes, have been extraordinarily prevalent, disabling them from work. These cases have been attended with but slight cough, no fever, considerable pain upon inspiration, and increased by the motion of the arms in walking. Bleeding, etc., have given the most decided relief. As is usual at this period of the year, we have many cases of pleurisy and pneumonia amongst the working poor, but which gave way to the usual treatment. Erysipelas has been more general than usual, I have never known it more so; whether arising from contagion is doubtful, as the cases appear to be isolated. Thirteen cases have come under my notice. During the last month, there have been great vicissitudes of temperature and more unequal atmospheric pressure, and the dew point higher than the average. This state has given rise to ague, remittent fever, and typhus, chiefly occurring in those who, from peculiarity of circumstances, are unable to resist these changes. They evidently do not arise from want of cleanliness, ventilation, or bad sewerage, but, in my opinion, from atmospheric causes. These have required the utmost attention, and although protracted in their course, have terminated favourably. From the same causes I attribute the numerous cases of diarrhœa which have occurred during the quarter, attacking all grades of society and accompanied by great abdominal pains, sickness, and smart fever; in children, three deaths have occurred. Two cases of puerperal peritonitis have come under notice, some days after delivery, which ultimately did well; and one case of phlegmasia dolens. Rheumatism has been more general than usual the last month, from exposure to cold and in those predisposed to the disease. This has given way to large doses of bicarbonate of potash, as recommended by Dr. Garrod. The casual diseases of the quarter are, hæmorrhage, paralysis, tic-douloureux, syphilis, dyspepsia, leucorrhœa, neuroses, consumption, and some cutaneous diseases."

***Record of Deaths extracted from the Registrar's Book.***

| *March.* | | *April.* | | *May.* | |
|---|---|---|---|---|---|
| Hydrocephalus .... | 1 | Hooping cough .... | 1 | Consumption ...... | 4 |
| Debility .......... | 1 | Debility .......... | 4 | Pneumonia........ | 1 |
| Dropsy............ | 1 | Convulsions ...... | 3 | Atrophy .......... | 3 |
| Paralysis.......... | 1 | Atrophy .......... | 1 | Decay of nature.... | 1 |
| Pneumonia........ | 1 | Consumption ...... | 1 | Hooping cough .... | 2 |
| Lying-in .......... | 1 | Dropsy .......... | 2 | Hydrothorax ...... | 1 |
| Atrophy .......... | 2 | Decay of nature .... | 1 | Debility .......... | 1 |
| Hooping cough .... | 2 | Puerperal fever .... | 1 | Diarrhœa ........ | 3 |
| Old age .......... | 1 | | | | |
| Aneurism ........ | 1 | | | | |
| Drowning ........ | 1 | | | | |
| Ossification of heart | 1 | | | | |
| Burn ........... | 1 | | | | |
| Kick from a horse .. | 1 | | | | |
| Scarlatina........ | 1 | | | | |
| Convulsions ...... | 1 | | | | |
| Bronchitis ........ | 3 | | | | |
| Accident .......... | 1 | | | | |
| | 22 | | 14 | | 16 |

*Wisbeach.* Mr. Hole writes :—" Hooping-cough, ague, and remittent fever have been very prevalent this quarter. There has also been more diarrhœa than is usual at this season of the year. The changes of temperature have been great and very sudden. The wind has been chiefly east and north-east. The district is not healthy now."

*Pontesbury.* Mr. Eddowes says :—" In the latter end of May, one family was seized with scarlet fever; six children had it. Before the youngest, a child at the breast, was seized, the mother had erysipelas of the face; one eye was swollen up. As she was getting better, the baby was seized with scarlet fever and erysipelas; one eye completely swelled up and the other nearly so. They all recovered."

*Nottingham.* Dr. Robertson writes :—The principal diseases here have been those involving the respiratory organs, measles, hooping-cough, and catarrh. The two former have been very fatal: forty-one persons having died from the former, and thirty-five from the latter, during the quarter. I subjoin the usual meteorological and disease report (for the former of which I am indebted to the courtesy of Mr. White of the General Hospital)."

| | *March.* | *April.* | *May.* |
|---|---|---|---|
| Barometer maximum | 30·37 | 30·01 | 29·92 |
| " minimum | 29·51 | 28·92 | 29·27 |
| " mean | 27·77 | 29·34 | 29·41 |
| Temperature maximum | 51·2 | 64·2 | 68·4 |
| " minimum | 29·8 | 31·3 | 31·5 |
| " mean | 40·6 | 45·3 | 50.3 |
| Total amount of rain | 0·70 | 1·11 | 1·26 |
| Prevailing direction of wind | N.E. & N. | SE. & E. | NE. & W. |
| Mean amount of ozone | 7 | 8 | 7 |
| New cases of disease | 345 | 298 | 268 |
| Number of deaths | 147 | 127 | 140 |

*Burton-on-Trent.* Dr. Spencer Thomson says :—" The quarter ending May 30th has, on the whole, been healthier than the previous one, the most prevalent diseases being catarrhs and bronchitic attacks during the east winds."

*Lincoln.* Mr. Lowe says :—" This quarter has been more than usually free from epidemic and endemic diseases; a few sporadic cases of scarlet fever, hooping-cough, influenza, croup, and erysipelas having occurred in April and May."

*Alford.* Dr. West says :—During the past quarter, I have had many cases of low pneumonia, complicated often with remittent fever and diarrhœa."

*Gainsborough.* Dr. Mackinder reports :—" There has been an epidemic of mumps this quarter, but of so mild a character as to require scarcely any medical treatment. Its infectious nature was clearly demonstrated in our workhouses, into which a woman and child were admitted with the disease on the 29th of April. On the 2nd of May, two children were affected; 3rd, eleven; 8th, two; 11th, one; 22nd, two. A few cases only of typhoid fever, acute rheumatism, and bronchitis have occurred in this locality, notwithstanding the cold north-easterly winds of March and April, and the cold, rain, and ozone of May."

The subjoined meteorological report has been furnished by Mr. Dyson:—"The months of March and April were remarkable for the dry state of the atmosphere and the cold, dry winds which prevailed. Only a quarter of an inch of rain fell in March, and two and a half inches in April. May was cold and damp; rain fell on twenty days, and the amount collected was nearly five inches. Ozone was very freely developed during May, the test-paper being deeply tinged nearly every day; the two previous months were as remarkable for the absence of ozone, except at distant intervals. The whole quarter was unusually cold, the first two months dry, and the last wet. A genial change occurred the first week in June. In consequence of the rain which fell in May, vegetation was early and luxuriant."

*Liverpool.*—Mr. Bickerton sends the following report:—"March 15th, 1856. During the last week there have been only 211 deaths registered. Diseases of the zymotic class caused 47 deaths. Of these there died from scarlatina 11; measles, 2; hooping-cough, 15; diarrhœa, 5; typhus, 4; small-pox, 1; syphilis, 1. Diseases of the lungs caused 75 deaths; 16 fewer than in last week. A female servant died at the age of 100. The temperature of the week has been very low, cold easterly winds prevailing.

"March 22, 1856. The number of deaths registered has been 251; an increase of 41 over last week. The increase in the week was owing in a great measure to the prevalence of easterly winds and colder weather, as well as to the increased number of deaths from scarlatina. Diseases of the lungs caused 96 deaths; scarlatina, 22; hooping cough, 13; diarrhœa, 8; measles, 7; typhus, 4; syphilis, 4; small-pox, 9. Gout produced one death; the patient was aged 66.

"March 29. There have been two hundred and thirteen deaths registered. Zymotic diseases caused fifty deaths. There were fewer deaths from diseases of the lungs than in last week, though easterly winds have prevailed with slight frosts at night; the variations, however, have been slight. The temperature for the week has been about one and a half degree below the average.

"During the quarter ending March 29, 1856, the number of deaths from all causes was 3,145, being 326 fewer than occurred in the same quarter of last year. They may probably be accounted for by the milder temperature in this than in last year. On the average the temperature in this quarter has been about six degrees higher than in the same quarter of last year. The comparative mortality in the two quarters shows a very material difference in the deaths from chest affections. In the first quarter of 1856 diseases of the chest caused 1685 deaths; while in the corresponding quarter of 1855 there were 2095 deaths—a difference of 410. Zymotic diseases caused this quarter 741 deaths, and in quarter just preceding 1093 deaths, there being a decrease in all diseases of this class, excepting pertussis, from which there has been a very great increase, causing 224 deaths, a larger number than in any previous quarter. The mortality from measles was 66, in the preceding quarter it was 153. Scarlatina also fell from 447 deaths in the preceding quarter, to 200; diarrhœa, from 112 to 65; typhus, from 96 to 60, being a less number than any previously known in Liverpool; the deaths from syphilis fell from 24 to 13; variola caused 17 deaths during the quarter.

"April 5. The deaths from all causes during the week have been 207; 239 being the average for the preceding eight years. The deaths from scarlatina have been 8; from measles, 8; from hooping-cough, 8; from typhus, 3; from erysipelas, 3; from tracheitis, 2; from diarrhœa, 1; from syphilis, 1; from small-pox, 1; diseases of the chest caused 81 deaths; intemperance, 2.

"April 12. The continued prevalence of fine mild weather exerts a very favourable influence upon the health of the town. The amount of sickness and death has been below the average; 211 deaths occurred in the borough, 225 being the average; scarlatina caused 7 deaths; measles, 10; hooping-cough, 16; typhus, 6; syphilis, 4; influenza, 1; small-pox, 1 (not previously vaccinated); diseases of the lungs caused 72 deaths.

"April 26. The weather during the past week has been very fine and seasonable, and the amount of disease has been less than usual. There were 194 deaths. Scarlatina caused 7 deaths; measles, 5; tracheitis, 6; hooping-cough, 5; typhus, 3; small-pox, 2; syphilis, 2; mumps, 1; purpura, 1; rheumatism, 3 (one, a girl ten years old; one male, twenty-three; and another forty-four years old); diseases of the lungs caused 68 deaths.

"May 3. There has been much unseasonable weather during the last seven days, it having been cold, with easterly winds prevailing. The number of deaths, however, has been considerably lower than the average, only twice during the last nine years has it been so low as in the past week. The total number of deaths in the borough during the week was 180, being 42 less than the corrected average for the preceding eight years. Scarlatina caused 6 deaths; measles, 3; typhus, 6; hooping-cough, 6; tracheitis, 4; diarrhœa or dysentery, none. Diseases of the lungs caused 73 deaths.

"May 10. There has been a continuance of cold disagreeable weather, with the wind fixed, either east or east north-east, much more like March than May weather. Hence most people have been complaining of common cold, chapped hands, and cracked lips. To day, however, true May weather has commenced. The number of deaths has been 180, being 40 below the average of the corresponding week in previous years. Scarlatina caused 4 deaths; measles, 7; typhus, 8; hooping-cough, 7; diarrhœa, 3; small-pox (not vaccinated), 1; syphilis, 6. Chest affections produced 56 deaths, viz., phthisis, 32; inflammatory affections of lungs, 24. Of the 180 deaths, 90 were males, 90 females; 83 adults, 97 children.

"May 17. The total number of deaths during the past week has been 178, the average being 222. Scarlatina caused 7 deaths; typhus, 6; hooping-cough, 6; diarrhœa, 3; measles, 2; syphilis, 1. Diseases of the lungs caused 75 deaths, there having been 56 last week. One death was caused by excessive drinking. Mean temperature, 52; average of preceding years, 53·7. Rain fell on four days.

"May 24. There have been during the past seven days 211 deaths registered in the borough, being 14 less than the corrected average, but 33 more than in the previous week. Scarlatina caused 13 deaths; measles, 9; typhus, 12 (being the greatest number in any week in the present year); hooping-cough, 6; diarrhœa, 6; small-pox, 2; syphilis, 2. There were 66 deaths from diseases of the lungs. A girl, six years old, died from sea sickness. There have been only three deaths registered in West Derby ward, with a population of 29,000.

"May 31. Mild weather has prevailed during the past week. There have been registered 181 deaths, being thirty fewer than in the preceding week. There were from scarlatina, 3 deaths; measles, 10; hooping-cough, 9; typhus, 5; small-pox, 3; diarrhœa, 4; syphilis, 1; English cholera, 1. Diseases of lungs caused 72 deaths. The temperature has been very mild and fine.

"The following is a return of all the inquests held before the borough coroner, P. F. Curry, Esq., during the year 1855. It will be seen that sudden deaths and deaths by violence occur more frequently amongst the uneducated than the educated classes. Of 1935 witnesses examined, only 681 were able to sign their names to the depositions.

| | |
|---|---|
| Accidentally killed | 159 |
| " scalded | 29 |
| " burnt | 51 |
| Excessive drinking | 34 |
| Injuries, how received no evidence | 23 |
| Children found exposed stillborn | 16 |
| Infanticide by persons unknown | 8 |
| Natural causes | 187 |
| Found drowned | 41 |
| Accidentally drowned | 21 |
| Suicide during temporary insanity | 14 |
| Felo-de-se | 4 |
| Children accidentally suffocated in bed | 65 |
| Wilful murder | 9 |
| Manslaughter | 8 |
| Other causes | 36 |
| Total | 705 |

| | |
|---|---|
| Number of inquests | 705 |
| Number of days on which inquests were held | 196 |
| Number of jurors who signed the inquisitions | 2352 |
| Number of jurors who wrote their own names | 3138 |
| Number of jurors who signed with their mark | 214 |
| Number of witnesses examined | 1935 |
| Number of witnesses who wrote their own names | 681 |
| Number of witnesses who signed with their mark | 1254 |

Expenses of witnesses, and car fares for jury, in the year 1855, £433 : 19 : 9.

*Bolton.*—Mr. Pendlebury says: "During the past quarter, cases of scarlet fever have been both fewer and milder; whilst those of measles have been evidently on the increase. There has been a tendency (as in the preceding quarter) to mucous inflammations, sometimes arising from apparently slight causes. Cases of erysipelas have been numerous. In two or three instances, the erratic form has been observed after vaccination of young infants. In other respects, the general health of the community has been satisfactory."

*York.*—Mr. Procter writes: "The cases of small-pox were, with one exception, in adults, all of whom had been vaccinated. Hooping-cough has been very prevalent and severe. The cases of influenza were characterised by neuralgic pains of the head, face, and neck; the bronchial symptoms were not severe. The cases of remittent fever were generally in young people. The mean temperature has been in March, 37·4; in April, 44·1; in May, 47·0."

*West Auckland.*—Mr. Todd says: "Many cases of abscess occurred during the progress of the epidemic of continued fever; they principally occurred in the back, and in the upper and lower extremities, and in malignant cases. This epidemic of continued fever arose from paludal sources, and in many cases was propagated by contagion. The fever that had its origin in *marsh miasmata* did affect others by infection or contagion."

*Rothbury.*—Mr. Summers writes: "In the cases of hooping-cough, all the patients were children who came from a distance for the sake of change of air while suffering from the disease. Though they mixed freely with other children who had never had hooping-cough, in no case did it spread to the latter. Throughout the quarter there has been a great tendency to asthenic complaints. Boils and abscesses have been of frequent occurrence. I have noted one case of actual carbuncle, and one of erysipelas, which ended in extensive sloughing of the skin and cellular tissue. The season in this district is very backward, with continuance of cold easterly winds and much rain."

